# Renewable synthesis of *n*-butyraldehyde from glucose by engineered *Escherichia coli*

**DOI:** 10.1186/s13068-017-0978-7

**Published:** 2017-12-04

**Authors:** Jason T. Ku, Wiwik Simanjuntak, Ethan I. Lan

**Affiliations:** 1 Department of Biological Science and Technology, National Chiao Tung University, 1001 Daxue Road, Hsinchu, 300 Taiwan; 2 Institute of Molecular Medicine and Bioengineering, National Chiao Tung University, 1001 Daxue Road, Hsinchu, 300 Taiwan

**Keywords:** Butanal, Butanol, CoA-acylating aldehyde dehydrogenase

## Abstract

**Background:**

*n*-Butyraldehyde is a high-production volume chemical produced exclusively from hydroformylation of propylene. It is a versatile chemical used in the synthesis of diverse C4–C8 alcohols, carboxylic acids, esters, and amines. Its high demand and broad applications make it an ideal chemical to be produced from biomass.

**Results:**

An *Escherichia coli* strain was engineered to produce *n*-butyraldehyde directly from glucose by expressing a modified *Clostridium* CoA-dependent *n*-butanol production pathway with mono-functional Coenzyme A-acylating aldehyde dehydrogenase (Aldh) instead of the natural bifunctional aldehyde/alcohol dehydrogenase. Aldh from *Clostridium beijerinckii* outperformed the other tested homologues. However, the presence of native alcohol dehydrogenase led to spontaneous conversion of *n*-butyraldehyde to *n*-butanol. This problem was addressed by knocking out native *E. coli* alcohol dehydrogenases, significantly improving the butyraldehyde-to-butanol ratio. This ratio was further increased reducing media complexity from Terrific broth to M9 media containing 2% yeast extract. To increase production titer, in situ liquid–liquid extraction using dodecane and oleyl alcohol was investigated. Results showed oleyl alcohol as a better extractant, increasing the titer of *n*-butyraldehyde produced to 630 mg/L.

**Conclusion:**

This study demonstrated *n*-butyraldehyde production from glucose. Through sequential strain and condition optimizations, butyraldehyde-to-butanol ratio was improved significantly compared to the parent strain. Results from this work may serve as a basis for further development of renewable *n*-butyraldehyde production.

**Electronic supplementary material:**

The online version of this article (10.1186/s13068-017-0978-7) contains supplementary material, which is available to authorized users.

## Background

Commodity chemical industry relies almost entirely on non-renewable petroleum and other fossil fuel resources, leading to environmental pollutions and the inevitable depletion crisis. As a potential solution, renewable chemical production through microbial conversion of biomass is an attractive direction for sustainability. However, chemicals natural to biological production are limited in quantity and type. Therefore, synthetic metabolic pathways are designed and engineered into microorganisms for increasing both the amounts and types of chemicals accessible to bio-based conversion.

**Table 1:** Strains and plasmids

Strain	Genotype	References
BW25113	*rrnB*_T14_ Δ*lacZ*_WJ16_ *hsd*R514 Δ*araBAD*_AH33_ Δ*rhaBAD*_LD78_	
XL1-blue	*recA1 endA1 gyrA96 thi*-*1 hsdR17 supE44 relA1 lac* [F’ *proAB lacI* ^*q*^ *ZΔM15 Tn10* (Tet^r^)]	Agilent Technologies
JCL16	BW25113/F’ [*traD36 proAB* ^+^ *lacI*qZΔM15 (Tet^r^)]	[30]
JCL299	JCL16 Δ*adhE* Δ*ldhA* Δ*frdBC* Δ*pta*	[9]
ELeco1	JCL299 Δ*yqhD*	This study
KS1	JCL299 Δ*yqhD* Δ*yjgB*	This study
KS2	JCL299 Δ*yqhD* Δ*yjgB* Δ*fucO*	This study
KS3	JCL299 Δ*yqhD* Δ*yjgB* Δ*fucO* Δ*eutG*	This study
KS4	JCL299 Δ*yqhD* Δ*yjgB* Δ*fucO* Δ*eutG* Δ*ybbO*	This study
KS5	JCL299 Δ*yqhD* Δ*yjgB* Δ*fucO* Δ*eutG* Δ*ybbO* Δ*adhP*	This study
KS6	JCL299 Δ*yqhD* Δ*yjgB* Δ*fucO* Δ*eutG* Δ*ybbO* Δ*adhP ΔgldA*	This study
KS7	JCL299 Δ*yqhD* Δ*yjgB* Δ*fucO* Δ*eutG* Δ*ybbO* Δ*adhP* Δ*gldA* Δ*yahK*	This study
KS8	JCL299 Δ*yqhD* Δ*yjgB* Δ*fucO* Δ*eutG* Δ*ybbO* Δ*adhP* Δ*gldA* Δ*yahK* Δ*yghA*	This study

Plasmid	Genotype	References
pRW13	P_ack_::*atoB, adhE2, crt, hbd*; ColE1 ori; Amp^r^	[19]
pRW18	P_adhE_::*fdh*; pSC101 ori; Cm^r^	[19]
pRW22	P_adhE_::*ter*; Cola ori; Kan^r^	[19]
pKU48	P_ack_::*atoB, aldh* (*Clostridium beijerinckii*), *crt, hbd*; ColE1 ori; Amp^r^	This study
pKU49	P_ack_::*atoB, aldh* (mutant gene; *Clostridium beijerinckii*), *crt, hbd*; ColE1 ori; Amp^r^	This study
pKU50	P_ack_::*atoB, aldh* (*Clostridium saccharobutylicum*)*, crt, hbd*; ColE1 ori; Amp^r^	This study
pKU51	P_ack_::*atoB, aldh* (*Clostridium saccharoperbutylacetonicum* N1-4)*, crt, hbd*; ColE1 ori; Amp^r^	This study

Gene sources are as follows: atoB, acetyl-CoA acetyltransferase from *E. coli*; adhE2, aldehyde–alcohol dehydrogenase from *Clostridium acetobutylicum;* crt, crotonase from *Clostridium acetobutylicum*; hbd, 3-hydroxybutyryl-CoA dehydrogenase from *Clostridium acetobutylicum*; fdh, formate dehydrogenase from *Candida boidinii, ter*, trans-enoyl-CoA reductase from *Treponema denticola I;* aldh, aldehyde dehydrogenase from sources indicated in the table

**Fig. 1:**
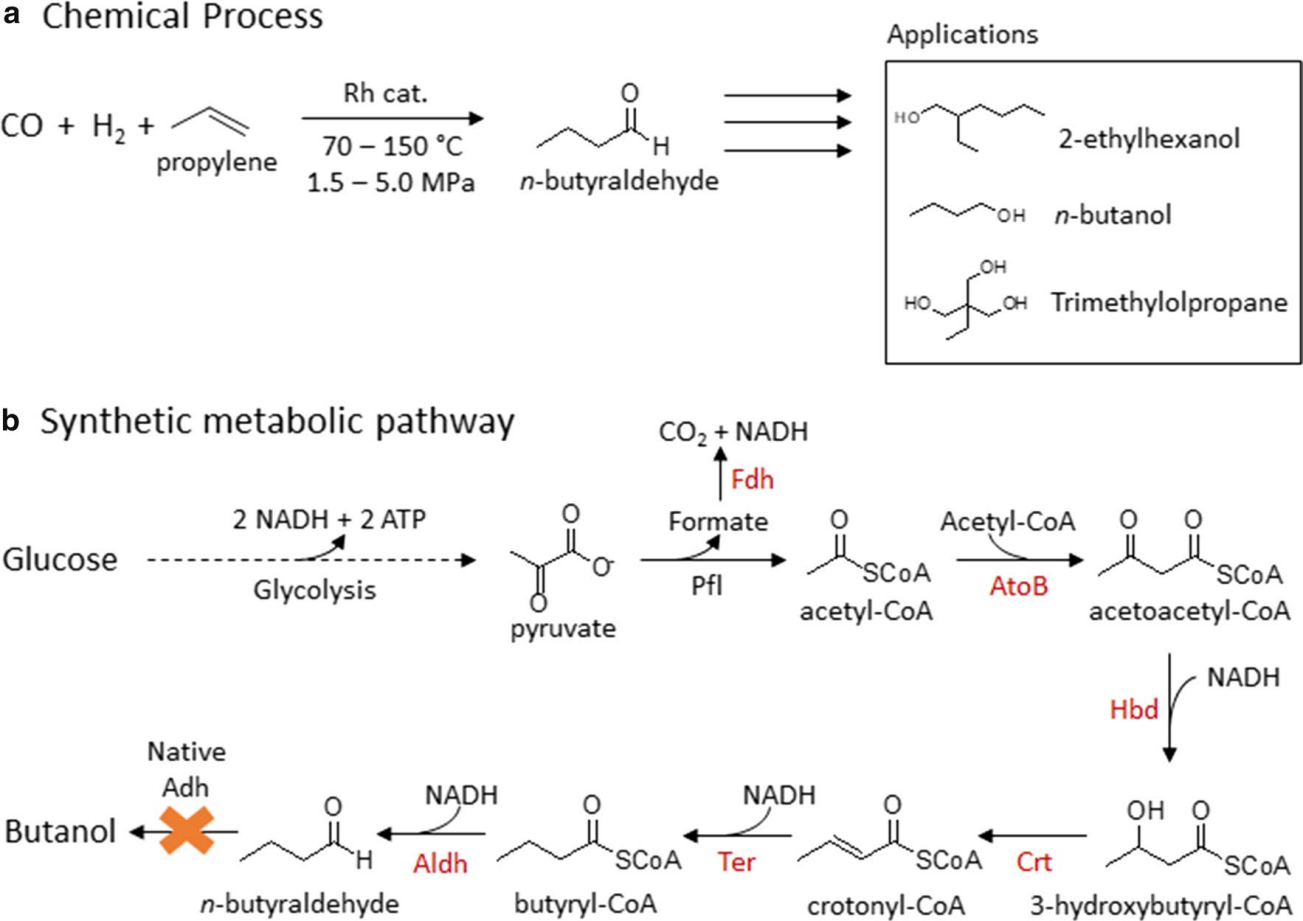
**a** Chemical synthesis of *n*-butyraldehyde from petrochemical feedstock. Propylene and syngas are reacted under high temperature and pressure to form *n*-butyraldehyde. Commercially important downstream products are shown as representative applications. **b** Metabolic pathway for *n*-butyraldehyde biosynthesis from glucose. Six genes are overexpressed to produce *n*-butyraldehyde. Native adh genes coding for alcohol dehydrogenases are knocked out to prevent excessive reduction of *n*-butyraldehyde. *Fdh* formate dehydrogenase; *AtoB* acetyl-CoA acetyltransferase; *Hbd* 3-hydroxybutyryl-CoA dehydrogenase; *Crt* crotonase; *Ter* trans-enoyl-CoA reductase; *Aldh* CoA-acylating aldehyde dehydrogenase

*n*-Butyraldehyde is a large volume chemical produced exclusively from hydroformylation of propylene (Fig. 1a) with annual production of 7 million tons and estimated growth of 2–3% per year [1]. As a reactive chemical, *n*-butyraldehyde is a versatile intermediate for the synthesis of various C4 and C8 alcohols, carboxylic acids, amines, and esters. In particular, 2-ethylhexanol has been a large volume derivative from *n*-butyraldehyde for its use in synthesizing phthalate plasticizers. Furthermore, *n*-butyraldehyde is also the precursor to polyvinyl butyral, a common polymer used for laminated glass in automotive and architectural industries. Recently, *n*-butyraldehyde has also been shown to be a biological precursor for bio-propane production [2]. These wide-spread applications of *n*-butyraldehyde make it an ideal chemical to be produced renewably.

Biological production of aldehydes is limited due to toxicity and reactivity. While few aldehyde products have been produced by engineered microbes [3–6], the biochemical repertoire for aldehydes needs to be expanded to support the effort in sustainability. *n*-Butyraldehyde has been previously reported in a mutant strain of *Clostridium acetobutylicum* [7] lacking alcohol dehydrogenase, capable of secreting up to 1.6 g/L of *n*-butyraldehyde. However, *Clostridia* are more difficult to work with than other well-characterized microorganism such as *Escherichia coli* and *Saccharomyces cerevisiae* due to their complex physiology and metabolism, as well as having less developed genetic manipulation tools. In addition, facultative anaerobes such as *E. coli* are often preferred for bio-based chemical productions because they grow rapidly during aerobic cultivation and conserves carbon for production under anaerobic conditions, increasing product yield due to elimination of respiration. Therefore, commercial interests are in engineering *E. coli* for *n*-butyraldehyde production [8]. However, due to the presence of numerous native alcohol dehydrogenases (Adh) in *E. coli*, *n*-butyraldehyde is spontaneously converted to *n*-butanol, thereby lowering the yield of aldehyde. This same behavior was observed in isobutyraldehyde production in *E. coli* [4]. Through knocking out several endogenous genes coding for Adh in *E. coli*, isobutyraldehyde production was significantly improved to roughly 2.5 g/L in test tubes and up to 35 g/L with gas stripping as in situ product removal. Inspired by the *E. coli* isobutyraldehyde production, here we also deleted *adh* genes and showed significant improvement in *n*-butyraldehyde production. Furthermore, in the process of constructing a *n*-butyraldehyde production pathway, we identified an alternative and better CoA-acylating aldehyde dehydrogenase than what has been previously reported. Lastly, instead of using gas stripping for in situ product removal as has been demonstrated for isobutyraldehyde, we tested in situ removal through organic overlay for liquid–liquid extraction and showed that oleyl alcohol is a suitable extractant for *n*-butyraldehyde production. The results obtained from this study provided a method for renewable synthesis of *n*-butyraldehyde with significantly reduced butanol co-production.

## Methods

### Strains and plasmids construction

Strains and plasmids used in this study are listed in the Additional file 1: Table S1. Primer sequences used are listed in Additional file 1: Table S1. Strains ELeco1 to KS8 were constructed from JCL299 [9] by sequential deletion of aldehyde reductase genes. All gene deletions were carried out using P1 transduction [10] with Keio collection [11] as donor strains. Kanamycin resistance marker was removed via FLP-mediated recombination. The successful gene deletions were subsequently verified by PCR (Additional file 1: Figure S1). All plasmids in this study were constructed using Gibson assembly [12]. Plasmids pKU48, pKU49, pKU50, and pKU51 were constructed by replacing *adhE2* in pRW13 with *aldh* (CB), *aldh* (CB(mut)), *aldh* (CS), and *aldh* (CS(N1-4)), respectively. Briefly, a fragment containing plasmid vector, *atoB*, *crt*, and *hbd* was amplified using primers KU115 and KU116 using pRW13 as a template. This fragment was assembled with individual aldh fragments amplified by the specified primers in Additional file 1: Table S1 using the genomic DNA of the corresponding source organism. aldh (CB(mut)) gene was cloned from a cloning vector containing the gene in our lab collection. For its sequence, see Additional file 1. Plasmids were then verified through sequencing.

### Culture media and growth conditions

All chemicals were purchased from Sigma-Aldrich or JTBaker. Media were purchased from BD-biosciences. All *E. coli* strains were cultured at 37 °C in a rotatory shaker (250 rpm). Luria broth (LB) and LB plates (1.5% w/v, agar) were routinely used for *E. coli* cultivation unless otherwise specified. Terrific broth (TB; 12 g tryptone, 24 g yeast extract, 2.31 g KH_2_PO_4_, 12.54 g K_2_HPO_4_, 4 mL glycerol per liter of water) supplemented with 20 g/L glucose was used as complex medium for *n*-butyraldehyde production. For medium analysis, M9 medium (12.8 g Na_2_HPO_4_·7H_2_O, 3 g KH_2_PO_4_, 0.5 g NaCl, 0.5 g NH_4_Cl, 1 mM MgSO_4_, 1 mg vitamin B1 and 0.3 mM CaCl_2_ per liter of water) supplemented with 20 g/L glucose and various concentrations (0.125–2.0%) of yeast extract (YE) and tryptone were used. When required, antibiotics were added into culture medium for selection at the following concentrations: kanamycin (Kan), 50 μg/mL; chloramphenicol (Cm), 50 μg/mL; ampicillin (Amp), 100 μg/mL; tetracycline (Tet), 15 μg/mL. Cell growth was routinely determined by measuring optical density at wavelength of 600 nm (OD_600_) of cultures using a Biotek epoch 2 microplate spectrophotometer. Path length was adjusted to 1 cm.

### *n*-Butyraldehyde production

1% (v/v) of overnight cultures in LB was used to inoculate 3 mL of production media (TB or M9 with varying concentration of yeast extract) containing 20 g/L glucose with appropriate antibiotics in test tubes. When the cultures reached OD_600_ of 0.4–0.6, they were switched to anaerobic by transferring them into a 10-mL BD vacutainer tube. Head space was then purged with anaerobic gas (95% N_2_, 5% H_2_). Cultures were then sampled at specified times for optical density measurement and product quantification.

### In situ removal of *n*-butyraldehyde by liquid–liquid extraction

1% (v/v) of overnight cultures were inoculated into 20 mL TB supplemented with 20 g/L glucose in 250-mL baffle flasks. When the culture OD600 reached 0.4–0.6, and 10 mL or 20 mL of either dodecane or oleyl alcohol was added as extractant to the culture. Subsequently, the cultures were switched to anaerobic by placing them in BD GasPak Anaerobe Gas Generating Pouch System with Indicator. When taking samples, anaerobic pouch was opened inside an anaerobic chamber to maintain the anaerobic environment.

### Product quantification

Culture samples (1 mL) were centrifuged at 10,000×*g* for 5 min. The supernatants were then collected for product analysis. When analyzing product content in extractant, extractant was diluted using ethyl acetate. Aldehyde and alcohol concentrations in both medium and extractant were quantified by a Shimadzu GC-2010 gas chromatography (GC) equipped with a barrier ionization discharge (BID) detector. The separation of compounds was performed by SH-Rtx-wax GC column (30 m, 0.32 mm i.d., 0.50-μm-thick film). GC oven temperature was initially held at 40 °C for 2 min and increased with a gradient of 5 °C/min until 80 °C followed by a gradient of 12 °C/min until 120 °C. Then the temperature continues to rise with a gradient of 20 °C/min until 230 °C and held for 2 min. Helium was used as the carrier gas. The injector was maintained at 220 °C, and the detector was maintained at 230 °C. 1 μL of samples was injected in split injection mode (1:15 split ratio) using 2-methyl-1-pentanol or 1-pentanol as the internal standard. Glucose consumption was determined by subtracting the glucose concentration in samples from the concentration in original medium. Glucose concentration was measured using Agilent 1260 HPLC equipped with a refractive index detector. The injection volume used was 20 μL. The mobile phase consisted of 5 mM H_2_SO_4_ with a linear flow rate of 0.6 mL/min. Separation of metabolites was done by Agilent HiPlex-H (700 × 7.7 mm) organic acid analysis column maintained at 65 °C. A Bio-Rad Micro-Guard Cation H guard column (30 × 4.6 mm) was connected in front of the analysis column. Glucose was monitored by refractive index detector. Concentration of glucose in the collected samples was determined by standard curve constructed from HPLC analysis of standard glucose solutions.

### Partition coefficient determination for *n*-butyraldehyde in dodecane and oleyl alcohol

To measure the partition coefficient of *n*-butyraldehyde for dodecane and oleyl alcohol, 800 μL of different concentrations of *n*-butyraldehyde (0.01, 0.02, 0.05, 0.1, 0.2% in water) were mixed with the same volume organic extractant in glass GC vials. The mixtures were sealed and mixed by vortex for 1 min followed by a 36-h incubation in 37 °C. After incubation, the concentrations of *n*-butyraldehyde in each phase were determined by GC as described in "Methods" section. The partition coefficients were calculated using the equation below:

$${\text{Partition coefficient}} = \log \frac{{\left[ {\text{Butyraldehyde}} \right]_{{\text{organic}}} }}{{\left[ {\text{Butyraldehyde}} \right]_{{\text{water}}} }}.$$

## Results and discussion

### Selection of CoA-acylating aldehyde dehydrogenase for *n*-butyraldehyde production

**Fig. 2:**
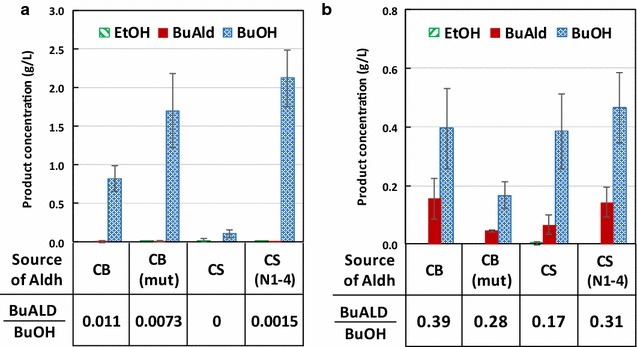
Production of butyraldehyde, butanol, and their ratios by strain **a** JCL299 and **b** ELeco1 expressing different Aldh with CoA-dependent pathway. CB, *C. beijerinckii*; CB (mut), mutant Aldh from *C. beijerinckii*; CS, *C. saccharobutylicum*; CS (N1-4), *C. saccharoperbutylacetonicum* N1-4; BuALD, *n*-butyraldehyde; BuOH, *n*-butanol. Error bars represent standard deviation of three experiments

*n*-Butyraldehyde is an intermediate in the *Clostridium* CoA-dependent *n*-butanol production pathway [9, 13, 14] (Fig. 1b). However, bifunctional aldehyde/alcohol dehydrogenase AdhE2 catalyzes the direct two-step conversion of butyryl-CoA to *n*-butanol, bypassing *n*-butyraldehyde as a product. To avoid conversion of *n*-butyraldehyde to *n*-butanol, we first replaced AdhE2 with CoA-acylating aldehyde dehydrogenase (Aldh), catalyzing only the conversion of butyryl-CoA to *n*-butyraldehyde. Some *Clostridia* such as *Clostridium beijerinckii* contain individual Aldh and Adh instead of a bifunctional enzyme for butanol production. Alternatively, Aldh is found in the degradation pathways for ethanolamine and 1,3-propanediol [15, 16]. However, the Aldh from ethanolamine and 1,3-propanediol utilization operons are not specific for butyryl-CoA reduction and have been previously shown to produce ethanol when expressed in *E. coli* [17]. Therefore, for the present study, we chose to work with *Clostridium* Aldh. Based on the sequence of *aldh* from *C. beijerinckii* [18], we selected two additional homologues from *C. saccharolyticum* and *C. saccharoperbutylacetonicum*, as well as a mutant *aldh* from *C. beijerinckii* which we isolated previously in our lab (see Additional file 1 for its sequence). These four *aldh* genes were individually cloned into synthetic operons with the genes necessary to convert acetyl-CoA to butyryl-CoA (Fig. 1b). These synthetic operons were driven by native *E. coli* promoter of *ack* and *adhE* genes, P_ack_ and P_adhE_, respectively, which have been previously shown to produce higher titers of butanol compared to using IPTG-inducible P_LlacO1_ promoter [19]. Here the P_ack_ and P_adhE_ were defined to include the ribosomal binding site and 5′ untranslated region upstream of their corresponding genes. *atoB*, *aldh*, *crt*, and *hbd* were cloned as one operon on a colE1 origin plasmid under the control of P_ack_. *ter* and *fdh* were individually expressed on colA and pSC101 origin plasmids, respectively, under the control of P_adhE_ [19]. These plasmids were transformed into *E. coli* strain JCL299 which was previously shown to efficiently produce *n*-butanol and has *ldhA*, *adhE*, *frdBC*, and *pta* knocked out [9]. Having the mixed acid fermentation pathways knocked out, JCL299 efficiently channels acetyl-CoA and NADH for the synthesis of *n*-butyraldehyde. As expected, due to the presence of endogenous alcohol dehydrogenases, the resulting strains showed minimal production of *n*-butyraldehyde (Fig. 2a). Majority of the fermentation products was *n*-butanol across the strains expressing the four different *aldh* genes. Chromosomal *yqhD*, coding for NADPH-dependent alcohol dehydrogenase, is known to reduce aldehydes to their corresponding alcohols and highly active as a detoxification mechanism [20–22]. Therefore, we knocked out *yqhD* in JCL299, yielding strain ELeco1. Expressing the *n*-butyraldehyde pathway in strain ELeco1, *n*-butyraldehyde production was observed. The best strain ELeco1/pKU48/pRW18/pRW22 produced 0.16 g/L of *n*-butyraldehyde (Fig. 2b). However, the aldehyde-to-alcohol ratio was 0.39. This low aldehyde-to-alcohol ratio indicates the presence of other active native Adh capable of reducing *n*-butyraldehyde.

### Improving aldehyde-to-alcohol ratio by knocking out native alcohol dehydrogenases

**Fig. 3:**
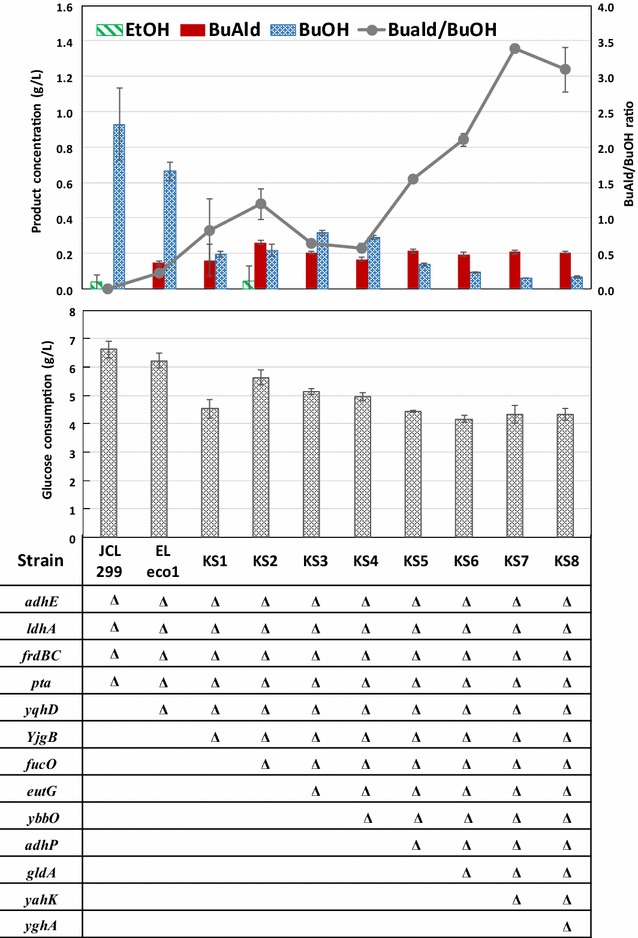
*n*-Butyraldehyde production and glucose consumption by different strains with alcohol dehydrogenase knock out in 24 h. All strains harbor pKU48, pRW18, and pRW22 for butytaldehyde production. For complete strain and plasmid list, see Table 1. Triangles in the table indicate gene knock out. Error bars represent standard deviation of three experiments

To decrease *n*-butanol formation and increase aldehyde-to-alcohol ratio, we knocked out the genes coding for other native Adh. Based on previous work for isobutyraldehyde production [23], we deleted eight *adh* genes that are likely to contribute to *n*-butyraldehyde reduction: *yjgB*, *fucO*, *eutG*, *ybbO*, *adhP*, *gldA*, *yahK*, and *yghA*. These genes were selected because their knockouts led to higher production titers of isobutyraldehyde. We sequentially knocked out each of these *adh* genes using P1 phage transduction with the Keio collection. Since all of these *adh* genes have been shown to be effective for increasing isobutyraldehyde production, they were knocked out without specific order. The results of *n*-butyraldehyde production titers and the aldehyde-to-alcohol ratio from these mutant strains are shown in Fig. 3. We noted that the titers achieved by the ELeco1/pKU48/pRW18/pRW22 are comparable to those achieved by the published patent application from Easel Biotechnologies [8], which used a strain with identical genotype. However, because the *n*-butanol titers were not reported in their work, the butyraldehyde-to-butanol ratios cannot be compared. Nevertheless, here we took the strain design further and showed that additional knockouts of native *adh* genes led to significant reduction in butanol. The final strain KS8/pKU48/pRW18/pRW22 reached an aldehyde-to-alcohol ratio of 3.1, representing an eightfold improvement compared to strain ELeco1/pKU48/pRW18/pRW22. Here we noted that strain KS7 harboring the same plasmids reached slightly higher aldehyde-to-alcohol ratio of 3.4. However, the difference in aldehyde-to-alcohol ratios was within error range and insignificant. Therefore, strain KS8 was used for downstream experiments. Among the eight additional *adh* that we knocked out, *eutG*, *ybbO*, and *yghA* showed no effect for increasing aldehyde-to-alcohol ratio. All other *adh* knock out contributed positively towards increasing aldehyde-to-alcohol ratio, indicating their native expression and corresponding enzymes’ capability of reducing *n*-butyraldehyde. While aldehyde-to-alcohol ratio increased with each *adh* gene knock out, the titer of *n*-butyraldehyde did not significantly increase. Correspondingly, glucose consumption by strains with each additional *adh* gene knock out also decreased (Fig. 3). These results indicate that carbon flux was reduced. Analysis of the thermodynamics of each step revealed that butyryl-CoA reduction to *n*-butyraldehyde is thermodynamically unfavorable with ΔG′° of 7.7 kJ/mol (calculated using eQuilibrator [24]), which may lead to inefficient conversion of butyryl-CoA to *n*-butyraldehyde, particularly after *n*-butyraldehyde concentration reached a certain threshold. It is likely that as *n*-butyraldehyde production is slowed, glucose metabolism also slows due to inability for NADH recycling. Since the native *E. coli* fermentation genes (*adhE*, *frdBC*, and *ldhA*) have been knocked out in strain KS8, *n*-butyraldehyde production becomes the only fermentative pathway available to recycle NADH back to NAD^+^. When *n*-butyraldehyde biosynthesis is slowed down, less NAD^+^ is available for use in glycolysis. As a result, glucose consumption rate decreases. Therefore, we next investigated the effect of in situ removal on *n*-butyraldehyde production titer.

### Improving *n*-butyraldehyde titer by in situ product removal

**Fig. 4:**
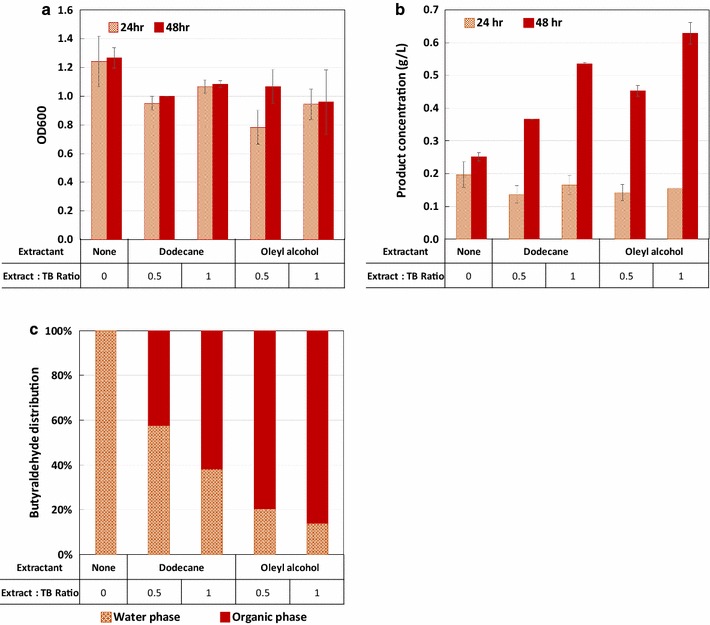
*n*-Butyraldehyde production using two-phase extraction for in situ product removal. **a** Cell growth in media with extractant dodecane and oleyl alcohol overlay. **b** Total *n*-butyraldehyde titer using different extractants. **c** *n*-Butyryaldehyde distribution in the culture media (water phase) and extractant (organic phase) for the 48-h samples. The ratio of extractant to TB is defined as the volume of extractant added divided by the 20 mL of culture (TB with 2% glucose). Error bars represent the standard deviation of triplicated experiments

Here we chose to use in situ liquid–liquid extraction for *n*-butyraldehyde removal using organic overlay. Dodecane and oleyl alcohol were selected as extractants because of their general application and non-toxicity to microbial cultures [25, 26]. We determined the partition coefficient of *n*-butyraldehyde in water and the two organic solvents by measuring the ratio of *n*-butyraldehyde appearing in both aqueous and organic phase (Additional file 1: Figure S2) after vigorous mixing followed by stationary incubation at 37 °C. The determined partition coefficient for *n*-butyraldehyde was 0.141 and 0.764 for dodecane and oleyl alcohol, respectively. This result indicated that oleyl alcohol may be a more suitable extractant for *n*-butyraldehyde as higher partition coefficient indicates the higher ratio of *n*-butyraldehyde found in the organic layer. These two extractants are only mildly toxic to *E. coli* as the growth of the *n*-butyraldehyde producing strain cultivated in the presence of either dodecane or oleyl alcohol were only slightly lowered compared to the control without any extractant (Fig. 4a), indicating the suitability of these solvents for in situ extraction. As results shown in Fig. 4b, *n*-butyraldehyde titer significantly improved in the presence of extractant. Consistently for both dodecane and oleyl alcohol, using 1 volume of extractant outperforms that using 0.5 volume. The best condition using oleyl alcohol with a 1:1 extractant-to-culture volume ratio produced over 0.6 g/L of *n*-butyraldehyde, representing a near three-fold improvement over no extractant. As expected, oleyl alcohol outperformed dodecane as extractant for *n*-butyraldehyde (Fig. 4c), consistent with the partition coefficients.

### Effect of reducing media complexity on *n*-butyraldehyde production

**Fig. 5:**
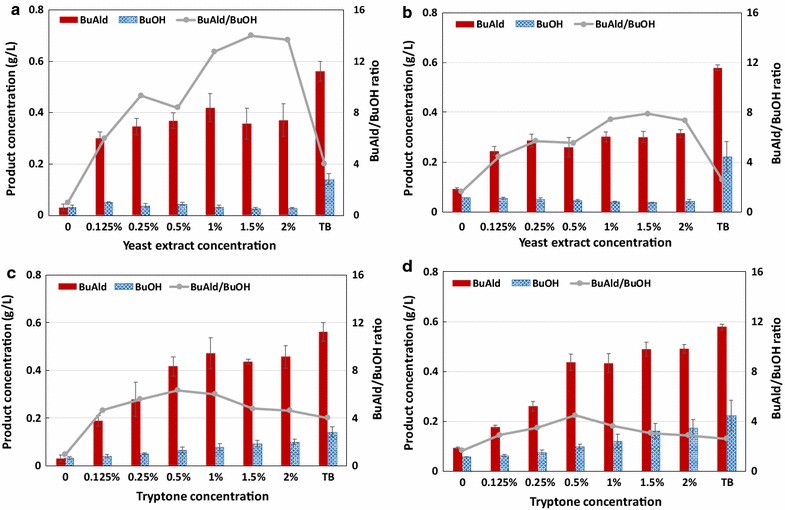
Comparison of yeast extract (**a**, **b**) and tryptone (**c**, **d**) concentration in M9 glucose media for *n*-butyraldehyde production. Product concentrations and butyraldehyde-to-butanol ratios sampled at **a**, **c** 24 h and **b**, **d** 48-h post-switch to anaerobic condition. Strain KS8/pKU48/pRW18/pRW22 was used for *n*-butyraldehyde production

Next, we evaluated the effect of yeast extract and tryptone concentration on *n*-butyraldehyde production. Using terrific broth (TB) is typically not commercially viable due to its expensive cost. Furthermore, aldehydes are reactive and can spontaneously form Schiff base with amines. Since TB contains high amounts of yeast extract and tryptone, aldehydes may likely be spontaneously reacted with the amino groups present on amino acids and oligopeptides in TB. Using M9 media with glucose as the base, we supplement 0 to 2% yeast extract or tryptone to determine an optimum level. Results are summarized in Fig. 5. Figure 5a shows the effect of yeast extract concentration on the production of *n*-butyraldehyde after 24 h of anaerobic incubation. *n*-Butyraldehyde titer was not significantly sensitive to yeast extract concentration between 0.125 and 2%. However, if no yeast extract was added, only minimal amount of *n*-butyraldehyde was observed, indicating the importance of complex nitrogen source. Interestingly, those using M9 with yeast extract had a higher aldehyde-to-alcohol ratio than that using TB due to higher levels of butanol produced. 48-h post-anaerobic switch (Fig. 5b) showed slight decrease in *n*-butyraldehyde and increase in *n*-butanol titer, indicating functional alcohol dehydrogenase actively converting *n*-butyraldehyde to *n*-butanol.

*n*-Butyraldehyde production was more sensitive to tryptone concentration than that of yeast extract as cultures containing 0.125 and 0.25% tryptone showed lower *n*-butyraldehyde titer compared to the corresponding concentrations of yeast extract (Fig. 5c, d). Increasing tryptone concentration led to increased *n*-butanol production, indicating that tryptone contributed towards the lowered aldehyde-to-alcohol ratio for using TB as production media. By comparing the components of yeast extract and tryptone from the manufacturers’ manual, we noticed that tryptone has higher percentage of larger molecules with molecular weight in the range of than 500–2000 Da, indicating a larger amount of oligopeptides. On the other hand, yeast extract contains mostly smaller molecules with molecular weight less than 250 Da. It is possible that this discrepancy led to different expression patterns which may include non-specific native alcohol dehydrogenases capable of reducing *n*-butyraldehyde. Nonetheless, the exact mechanism to why tryptone causes increase in *n*-butanol production is unclear.

## Conclusions

This study demonstrated *n*-butyraldehyde production from glucose using engineered *E. coli*. We showed that *aldh* gene from *C. beijerinckii* outperformed the other *aldh* genes tested in achieving highest butyraldehyde-to-butanol ratio. Subsequent knockouts of endogenous *adh* genes including *yqhD*, *yjgB*, *fucO*, *adhP*, *gldA*, and *yahK*, in situ product removal by oleyl alcohol, and medium optimization using M9 2% glucose with 1–2% yeast extract significantly improved both the *n*-butyraldehyde titer (from 10 mg/L to 630 mg/L) and butyraldehyde-to-butanol ratio. Compared to *E. coli* glucose-based *n*-butanol production (with titer up to 15 g/L in test tubes), *n*-butyraldehyde production using similar strain and pathway resulted in significantly lower titer. It is possible to achieve renewable *n*-butyraldehyde production via bio-butanol followed by chemical conversion. The chemical conversion of *n*-butanol to *n*-butyraldehyde is possible using Cu [27, 28]- or Pt [29]-based catalysis. However, the Cu-based catalysis requires high temperature of 500 to 800 K. While the Pt-based catalysis can produce *n*-butyraldehyde from *n*-butanol at lower temperatures, leaching of the expensive Pt-based catalyst increases cost of the overall process. Therefore, sugar-based direct production of *n*-butyraldehyde remains an attractive potential direction. In order for it to become industrially viable in the future, further optimization of genetic expression, media, and product removal techniques is necessary.

## Additional file

**Additional file 1.** Supporting information.
