# Supplementary material for: Renewable synthesis of n-butyraldehyde from glucose by engineered Escherichia coli
Source: Biotechnol Biofuels. 2017 Dec 4;10:291. doi: 10.1186/s13068-017-0978-7 (PMC5713646; doi:10.1186/s13068-017-0978-7)
Supplement: Supplementary file 1 — Additional file 1. Supporting information. [file 13068_2017_978_MOESM1_ESM.docx]

**Additional file**

Renewable synthesis of n-butyraldehyde from glucose by engineered *Escherichia coli*

Jason T. Ku,^[b]^ Wiwik Simanjuntak, ^[a]^ and Ethan I. Lan *^[a]^

[a] W. Simanjuntak, Prof. E.I. Lan
Department of Biological Science and Technology
National Chiao Tung University
1001 Daxue Road, East District, Hsinchu City, 300
E-mail: ethanilan@nctu.edu.tw

[b] J.T. Ku
Institute of Molecular Medicine and Bioengineering
National Chiao Tung University
1001 Daxue Road, East District, Hsinchu City, 300

**DNA sequence of *aldh* (*CB*(mut))**

ATGAATAAAGACACACTAATACCTACAACTAAAGATTTAAAAGTAAAAACAAATGGTGAAAACATTAATTTAAAGAACTACAAGGATAATTCTTCATGTGTCGGAGTTTTCGAAAATGTTGAAAATGCTATAAGCAGCGCTGTACACGCACAAAAGATATTATCCCTTCATTATACAAAAGAGCAAAGAGAAAAAATCATAACTGAGATAAGAAAGGCCGCATTACAAAATAAAGAGGTCTTGGCTACAATGATTCTAGAAGAAACACATATGGGAAGATATGAGGATAAAATATTAAAACATGAATTGGTAGCTAAATATACTCCTGGTACAGAAGATTTAACTACTACTGCTTGGTCAGGTGATAATGGTCTTACAGTTGTAGAAATGTCTCCATATGGTGTTTTAGGTGCAATAACTCCTTCTACGAATCCAACTGAAACTGTAATTTGTAATAGCATAGGCATGATAGCTGCTGGAAATGCTGTAGTATTTAACGGACACCCATGCGCTAAAAAATGTGTTGCATTTGCTGTTGAAATGATAAATAAGGCAATTATTTCATGTGGCGGTCCTGAAAATCTAGTAACAACTATAAAAAATCCAACTATGGAGTCTCTAGATGCAATTATTAAGCATCCTTCAATAAAACTTCTTTGCGGAACTGGGGGTCCAGGAATGGTAAAAACCCTCTTAAATTCTGGTAAGAAAGCTATAGGTGCTGGTGCTGGAAATCCACCAGTTATTGTAGATGATACTGCTGATATAGAAAAGGCTGGTAGGAGCATCATTGAAGGCTGTTCTTTTGATAATAATTTACCTTGTATTGCAGAAAAAGAAGTATTTGTTTTTGAGAATGTTGCAGATGATTTAATATCTAACATGCTAAAAAATAATGCTGTAATTATAAATGAAGATCAAGTATCAAAATTAATAGATTTAGTATTACAAAAAAATAATGAAACTCAAGAATACTTTATAAACAAAAAATGGGTAGGAAAAGATGCAAAATTATTCTTAGATGAAATAGATGTTGAGTCTCCTTCAAATGTTAAATGCATAATCTGCGAAGTAAATGCAAATCATCCATTTGTTATGACAGAACTCATGATGCCAATATTGCCAATTGTAAGAGTTAAAGATATAGATGAAGCTATTAAATATGCAAAGATAGCAGAACAAAATAGAAAACATAGTGCCTATATTTATTCTAAAAATATAGACAACCTAAATAGATTTGAAAGAGAAATAGATACTACTATTTTTGTAAAGAATGCTAAATCTTTTGCTGGTGTCGGTTATGAAGCAGAAGGATTTACAACTTTCACTATTGCTGGATCTACTGGTGAGGGAATAACCTCTGCAAGGAATTTTACAAGACAAAGAAGATGTGTACTTGCCGGCTAA

**Table S1.** Primers used for plasmid construction

| Primer | Oligonucleotides(5’ -> 3’) |
| --- | --- |
| KU115-crt-F | TAATCTAGAAGGAGATATACCATGGAACTAAACAA |
| KU116-atoB-R | CATGGTACCTTTCTCCTGCATGCTTAAT |
| KU119-CBaldh-F | GCATGCAGGAGAAAGGTACCATGAATAAAGACACACTAATACCTACAACTAAAGATTT |
| KU120-CBaldh-R | ATGGTATATCTCCTTCTAGATTAGCCGGCAAGTACACATCTTCTTT |
| KU121-CBaldh(mut)-F | GCATGCAGGAGAAAGGTACCATGAATAAAGACACACTAATACCTACAACTAAAGATTTA |
| KU122-CBaldh(mut)-R | ATGGTATATCTCCTTCTAGATTAGCCGGCAAGTACACATCTTCTTT |
| KU123-CSaldhA-F | GCATGCAGGAGAAAGGTACCATGAATAATAATTTATTCGTGTCACCAGAAACTAA |
| KU124-CSaldhA-R | ATGGTATATCTCCTTCTAGATTAGCCTACGAACACACACCTTCTTTGT |
| KU125-N1_4-F | GCATGCAGGAGAAAGGTACCATGATTAAAGACACGCTAGTTTCTATAACAAAAGA |
| KU126-N1_4-R | ATGGTATATCTCCTTCTAGATTAACCGGCGAGTACACATCTTCTTT |


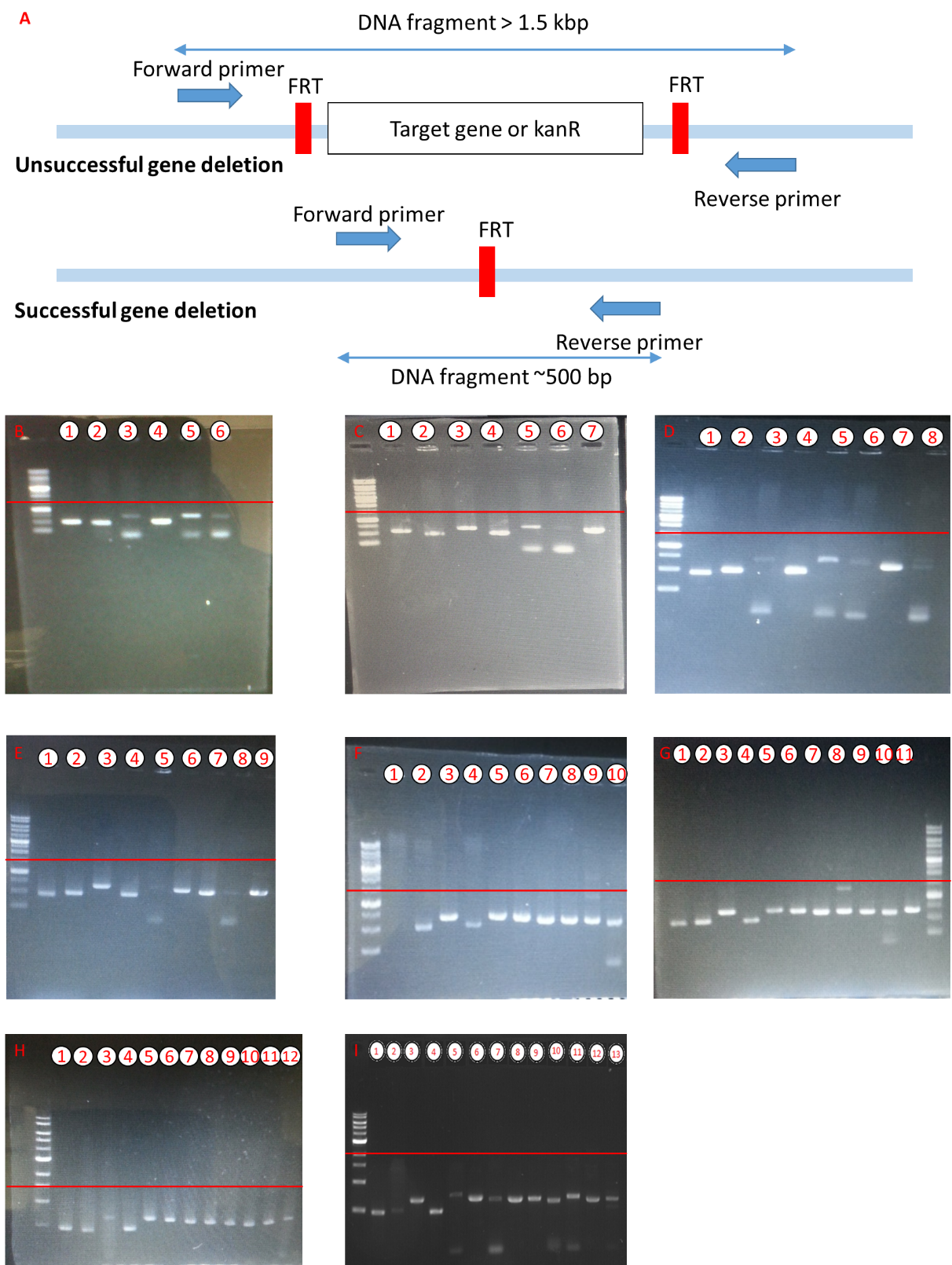


**Figure S1.** Verification of aldehyde reductase deleted strain by colony PCR. (A) Scheme of primer design for successful gene deletion verification. The gel pictures (B)-(I) corresponded to the strain KS1-KS8, respectively. The lanes labeled 1-13 represent the successful genes deletion of *adhE, ldhA, frdBC, pta, yqhD, yjgB, fucO, eutG, ybbO, adhp, glda, yahk*, and *yghA*, respectively. The bands of successful gene deletion verification should be around 500 bps while the unsuccessful strain showed a band more than 1.5 kbp. The red line in gel pictures represents the position of 1.5 kbp DNA fragment.


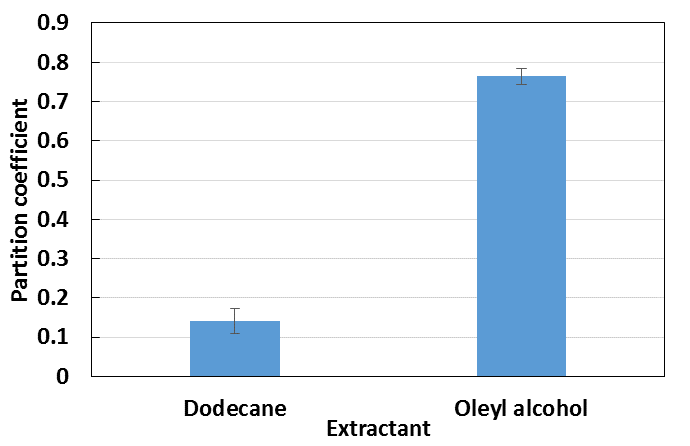
**Figure S2.** Partition coefficient for n-butyraldehyde in mixture of water with dodecane and oleyl alcohol individually. To measure the partition coefficient of *n*-butyraldehyde for dodecane and oleyl alcohol, 800 μL of different concentrations of *n*-butyraldehyde (0.01%, 0.02%, 0.05%, 0.1%, 0.2% in water) were mixed with the same volume organic extractant in glass GC vials. The mixtures were sealed and mixed by vortex for 1 minute followed by a 36-hour incubation in 37 °C. After incubation, the concentrations of *n*-butyraldehyde in each phase were determined by GC as described in the materials and methods section. The partition coefficients were calculated using equation below:

$$Partition coefficient=\log\frac{{[Butyraldehyde]}_{organic}}{{[Butyraldehyde]}_{water}}$$
